# Towards a New, Endophenotype-Based Strategy for Pathogenicity Prediction in BRCA1 and BRCA2: In Silico Modeling of the Outcome of HDR/SGE Assays for Missense Variants

**DOI:** 10.3390/ijms22126226

**Published:** 2021-06-09

**Authors:** Selen Özkan, Natàlia Padilla, Xavier de la Cruz

**Affiliations:** 1 Research Unit in Clinical and Translational Bioinformatics, Vall d’Hebron Institute of Research (VHIR), Universitat Autònoma de Barcelona, 08035 Barcelona, Spain; selen.ozkan@vhir.org (S.Ö.); natalia.padilla@vhir.org (N.P.); 2 Institució Catalana de Recerca i Estudis Avançats (ICREA), 08010 Barcelona, Spain

**Keywords:** BRCA1, BRCA2, endophenotype, breast cancer, functional assays, pathogenicity predictions, homology-directed DNA repair (HDR), molecular diagnosis, ovarian cancer, protein-specific predictor

## Abstract

The present limitations in the pathogenicity prediction of BRCA1 and BRCA2 (BRCA1/2) missense variants constitute an important problem with negative consequences for the diagnosis of hereditary breast and ovarian cancer. However, it has been proposed that the use of endophenotype predictions, i.e., computational estimates of the outcomes of functional assays, can be a good option to address this bottleneck. The application of this idea to the BRCA1/2 variants in the CAGI 5-ENIGMA international challenge has shown promising results. Here, we developed this approach, exploring the predictive performances of the regression models applied to the BRCA1/2 variants for which the values of the homology-directed DNA repair and saturation genome editing assays are available. Our results first showed that we can generate endophenotype estimates using a few molecular-level properties. Second, we show that the accuracy of these estimates is enough to obtain pathogenicity predictions comparable to those of many standard tools. Third, endophenotype-based predictions are complementary to, but do not outperform, those of a Random Forest model trained using variant pathogenicity annotations instead of endophenotype values. In summary, our results confirmed the usefulness of the endophenotype approach for the pathogenicity prediction of the BRCA1/2 missense variants, suggesting different options for future improvements.

## 1. Introduction

Our ability to identify the risk variants in the BRCA1 and BRCA2 (BRCA1/2) proteins is a valuable source of information about hereditary cancer patients. For example, it is routinely used in the clinical management of hereditary breast and ovarian cancer (HBOC) syndrome patients [1] in processes like counseling about preventive surgeries [2,3,4], mastectomy or salpingo-oophorectomy, channeling carriers to surveillance programs, etc. In metastatic castration-resistant prostate cancer, BRCA2 mutations are related to more aggressive forms of the disease and prepare us for worse patient survival rates [5]. From the point of view of therapy, finding germline variants in these proteins is also relevant, e.g., recent results indicate that BRCA1/2 testing helps design the treatment strategy for breast and ovarian cancer patients [3,4,6]. More precisely, we know that carriers of BRCA1/2 high-risk variants can benefit from synthetic lethal therapies [7], like the use of a poly(ADP-ribose) polymerase (PARP) inhibitor [8,9]. Recent evidence indicates that the same will be the case for prostate cancer patients [5].

The previous considerations underlined the significance of being able to discriminate between benign and pathogenic BRCA1/2 missense variants. In silico tools, like Align-GVGD [10], SIFT [11], Polyphen-2 [12], etc., provide a fast, non-expensive approach to address this problem. Although, with their present performances, these tools are not recommended as stand-alone sources of evidence [13,14], it is clear that the recent surge of new ideas and techniques in the prediction field [15] may change this situation sooner than expected. A good illustration of these developments can be found in the results of the CAGI 5 international challenge, organized to assess the state-of-the-art developments in the field of variant interpretations [16].

In general, in silico tools address the variant interpretation problem as a binary classification problem in which variants are characterized with a series of features used to decide on the pathogenic nature of the amino acid replacement [15,17]. While valuable results are obtained with this approach, it also has some limitations. First, they generally integrate heterogeneous information from different sources that may bias the predictions due to unknown redundancies. Second, the results of most standard pathogenicity predictors are hard to interpret, due to either the machine learning technology employed or to the properties utilized to characterize variants, e.g., in the case of metapredictors [15]. This is problematic, because interpretation is a key aspect in the design of software tools for healthcare applications [18], particularly when they are not 100% accurate. Third, conventional in silico tools are limited in the sense that they do not produce mechanistic views of the impacts of variants and, consequently, are of little help in the relevant processes such as drug design.

The need to solve these challenges, as well as the plateau reached in the performances of silico tools in previous years [17], is behind the recent flurry of ideas in the field. Among these, it is worth mentioning a conceptual change in the prediction goal, in which the prediction objective (the binary, pathogenic/benign, clinical phenotype) would be replaced by the prediction of a mutant endophenotype [19]. An endophenotype refers to [19] “… quantitative measurements that are correlated with phenotypes via shared genetic causes (e.g., enzyme catalytic activity, serum cholesterol or glucose level, volumetric lung capacity)”. A priori, it is easier to predict than a clinical phenotype, because it is closer to the genotype, i.e., less effects from the genetic background are expected to intervene. Additionally, the change in the prediction objective, from a clinical phenotype to an endophenotype, is coherent with the continuous nature of the disease, itself naturally related to the continuous value associated with the functional changes promoted by mutations [20]. From a clinical point of view, focusing on endophenotypes does not necessarily mean a loss of value, e.g., in the case of BRCA1, Findlay et al. [21] established that there is a good correlation between the endophenotype (in their case, the values of the saturation genome editing, SGE, assay) and the binary (pathogenic/benign) clinical phenotypes of variants. That is, an endophenotype could a priori be used for variant annotation in the clinical setting. In addition, working with endophenotype estimates may (i) generalize the applicability of the predictions, which would be less dependent on the factors such as ethnicity/genetic background, (ii) provide a source of evidence easier to integrate into the diagnostic guidelines and (iii) contribute to the mechanistic interpretation of the impacts of the variants. However, when contemplating the applicability of endophenotype estimates to the clinical setting, we need to establish their accuracy, as it may vary depending on the assay considered. For example, we expect differences between predicting the results of homology-directed DNA repair (HDR) assays and volumetric lung capacities. A second aspect that we need to consider is whether the accuracy of endophenotype estimates translates to competitive pathogenicity phenotype predictions.

We recently explored these issues in the case of BRCA1/2 variants, developing a simple multiple linear regression (MLR) model to estimate the results of the HDR assays on these proteins [22]. We participated in the CAGI 5-ENIGMA challenge with this method, predicting the clinical phenotypes of the 144 BRCA1 and 174 BRCA2 variants. The results obtained were promising (our MLR model ranked second after LEAP [23]), supporting the use of endophenotype estimates for predicting the pathogenic nature of variants. However, due to the nature of the CAGI challenge, to which only a limited number of results can be submitted and, also, to the limitations in the datasets employed, we decided to further test this approach.

The goal of the present work was to explore more closely, in the case of BRCA1/2, the use of in silico endophenotype estimates for the interpretation of missense variants. Using as starting points the MLR model that we presented at the CAGI challenge [22] and the outcome of the HDR [24,25] and SGE [21] assays as target endophenotypes, we characterized the effects of different terms (better description of the amino acid properties and the role of multiple sequence alignments) in the accuracy of endophenotype estimates. Then, we studied if the accuracy of the resulting models was good enough to generate competitive pathogenicity predictions. To this end, we compared the performances of these models with that of a set of ten public (like REVEL [26], Align-GVGD [10], etc.) and two in-house (a Random Forest and a Neural Network classifier) in silico tools in the pathogenicity prediction of the BRCA1/2 variants of the known clinical phenotype. After describing the resulting data, we discussed the relevance of the endophenotype estimates for the pathogenicity prediction and how we can advance in their improvement.

## 2. Results

First, we explored to which extent we can estimate computationally, using simple properties, the impact of missense variants on two endophenotypes related to the BRCA1/2 proteins and the outcome of the HDR and SGE functional assays. Then, we have studied if, after binarization, the endophenotype estimates could result in pathogenic/benign predictions comparable in accuracy to those of the standard in silico tools. These analyses are divided in the following sections.

First, we studied how well the predictive properties’ chosen model endophenotype changes upon mutation for the HDR and SGE assays. Since conservation-based measures (Shannon’s entropy and position-specific scoring matrix) depend on the multiple sequence alignment (msa) used, we tested the robustness of our results using two different msa, one constituted by the orthologs of BRCA1/2 (Omsa) and the other by the homologs of BRCA1/2 (Hmsa) (see Materials and Methods). The results shown in the figures generally correspond to Omsa, while those for Hmsa, as well as a few Omsa results, are presented in the Supplementary Materials.

Second, for a set of variants with known pathogenic/benign annotations, we tested the success rates of the endophenotype estimates when used for pathogenicity predictions. To this end, for these variants, we transformed the HDR and SGE estimates obtained with our regression models into pathogenicity predictions that we then compared with the known clinical phenotype annotations.

Finally, we compared the previous procedure for obtaining pathogenicity predictions against (i) a set of ten representative in silico tools and (ii) two in-house methods, Random Forest (RF) and a neural network (NN) that used as input features the five variants utilized to build the regression models.

### 2.1. Modeling the Impact of BRCA1/2 Variants on Endophenotype

**Figure 1:**
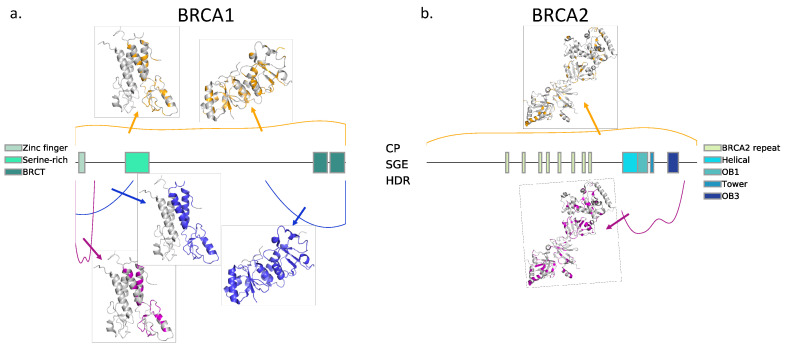
The sets of BRCA1/2 variants used in this work. The figure shows, for (**a**) BRCA1 and (**b**) BRCA2, the distributions of the variants for the Homology-Directed Repair (HDR, pink), Saturation Genome Editing (SGE, blue) and Clinical Phenotype (CP, orange) datasets along the sequence of these proteins. Functional domains are shown using rectangular boxes. We can see that, for both proteins, the variants with known endophenotype values (i.e., results of the functional assays HDR and SGE) concentrate at the N- and C-terminal domains. On the contrary, those variants of known pathogenic nature (benign/pathogenic; CP dataset) are distributed along the whole sequence. For those cases for which it was possible, we also showed the location of the variants in the three-dimensional structures available (BRCA1, PDB codes: 1jm7 and 1t15; BRCA2, PDB code: 1iyj).

We chose, as endophenotypes, two functional assays whose values were available in the literature (see Materials and Methods): HDR for BRCA1 [24] and BRCA2 [25] and SGE for BRCA1 [21]. In Figure 1, we describe the sets of variants for which the results of HDR and SGE are available. The output of these assays was a continuous variable, and, extending our previous work [22], we used regression tools for modeling its value. We tested five different types of regressions for this purpose (see Materials and Methods): MLR (based on our original model [22]), Ridge, Lasso, Elastic and Kernel. Here, we describe the performance of these models. In Table 1, we describe the combinations of HDR and SGE data used for training/testing purposes.

#### 2.1.1. Predicting the Outcome of HDR Assays Using MLR

**Figure 2:**
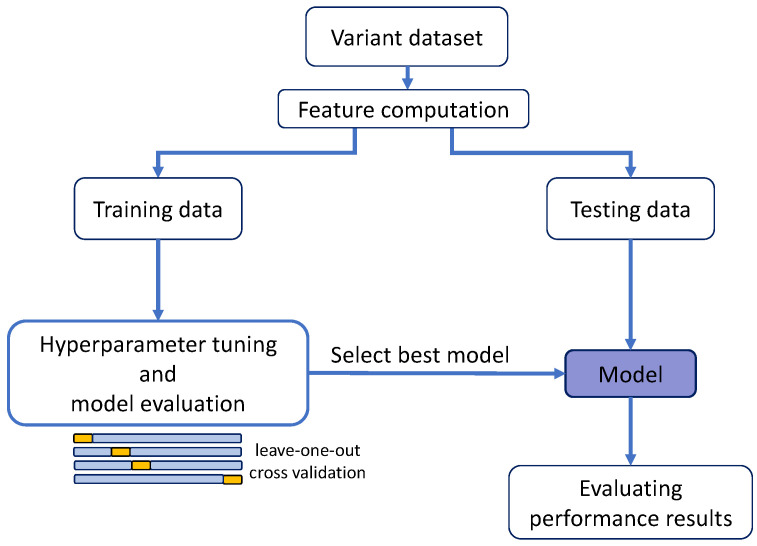
Scheme used to develop the regression models for obtaining the endophenotype (functional assay) estimates. The boxes represent the main steps in the process, which include the obtention of the variant dataset (a set of variants with available endophenotype values), the characterization of the variants with predictive features, etc. An important point in this scheme is the splitting of the data into two datasets, a training and a test dataset, required to avoid overfitting in the training of the regression models. When applied, the resampling step mentioned in the Discussion was executed immediately after the building of the variant dataset.

**Figure 3:**
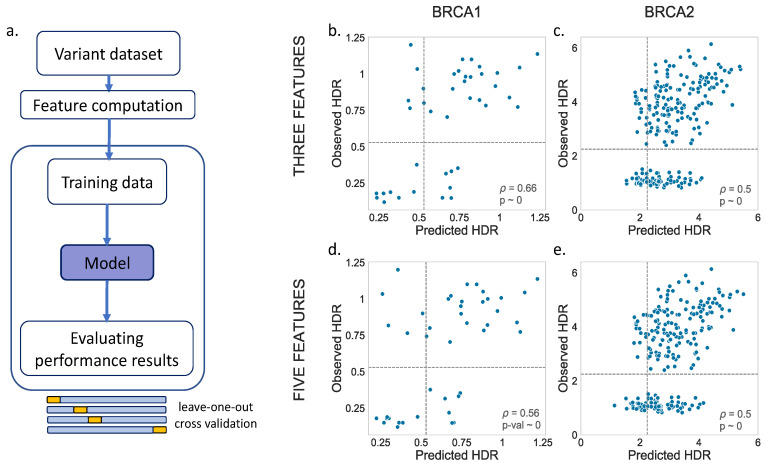
Observed vs. Predicted endophenotypes for the MLR model. When the endophenotypes are represented using the HDR functional assays, the small amount of data available for training purposes may prevent the training of the regularized models, like the Ridge, Lasso and Elastic. In this situation, that only affects BRCA1, we applied a training alternative to the scheme in Figure 2, following a rigorous LOOCV procedure (**a**). The results were obtained for two regression models: one with three ((**b**) BRCA1 and (**c**) BRCA2) and another with five input features ((**d**) BRCA1 and (**e**) BRCA2). The values of *p*, the Spearman’s Rank correlation, together with the corresponding *p*-values, are shown at the bottom-right of the figure. Note that the BRCA2 results are shown only for completeness, because there are enough variants for this protein to train the predictors according to the scheme in Figure 2. The dashed lines indicate the functionality thresholds, taken from the literature in the field and used in this work to binarize the endophenotype estimates into benign/pathogenic classes.

For BRCA1, the variants with HDR data available (Table 1), 44, were too few to split them into training and test sets, following the scheme in Figure 2 (a requirement for the regularization methods). For this reason, we only generated the MLR model; the other regression models were not derived. The performance of this MLR was estimated following a standard leave-one-out cross-validation (LOOCV) (Figure 3a). For BRCA2, we applied the same process for comparison, although, in this case, as we will see later, the larger variant dataset allowed us to apply a splitting procedure.

For the MLR models, we tried two options, one with three (Blosum62, Shannon’s entropy and a position-specific scoring matrix) and another with five (Blosum62, Shannon’s entropy, a position-specific scoring matrix, van der Waals volume and hydrophobicity) predictive features. The results are shown in Figure 3b,d (BRCA1) and Figure 3c,e (BRCA2). We noticed a moderate relationship between the observed and predicted values for both BRCA1 (three feat.: *p* = 0.66, *p*-value ~ 0; five feat.: *p* = 0.56, *p*-value ~ 0) and BRCA2 (three feat.: *p* = 0.50, *p*-value ~ 0; five feat.: *p* = 0.50, *p*-value ~ 0). A visual inspection showed no substantial differences between the models with three vs. five features.

In summary, we found that, for BRCA1/2, and within the limits imposed by our datasets, we can use the chosen features to estimate the change of an endophenotype upon a mutation with moderate accuracy.

#### 2.1.2. Predicting the Outcome of BRCA1/2 Variants with Other Regression Models

Here, we worked with other combinations of training/test data (Table 1) that allowed a full application of the scheme in Figure 2. After fitting the regression models to the training set, their performances were validated in the test set, comparing the observed and predicted values. This procedure was followed for the MLR, Ridge, Lasso, Elastic and Kernel regressions.

**Figure 4:**
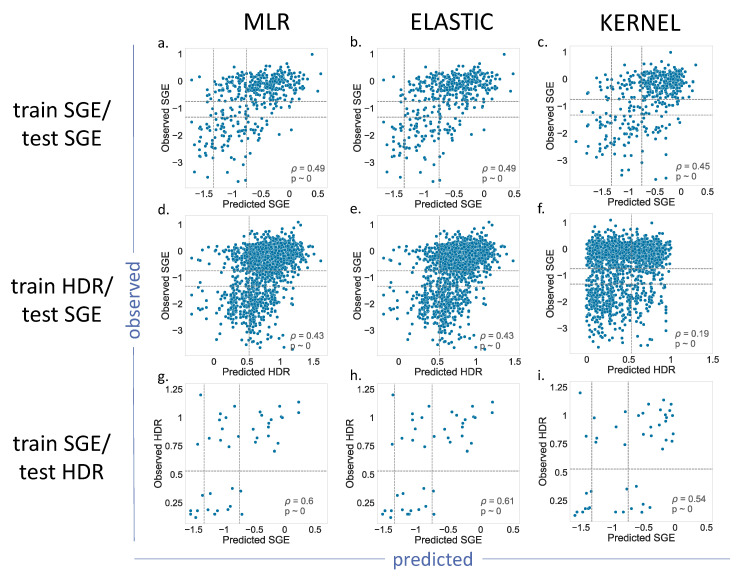
Observed vs. Predicted endophenotypes for the (**a**,**d**,**g**) MLR, (**b**,**e**,**h**) Elastic and (**c**,**f**,**i**) Kernel regressions. BRCA1 case. Each row corresponds to the results obtained with a specific combination of training/test datasets. For example, for the train SGE/test SGE, the dataset was split into a training and a test dataset containing 75% and 25% of the variants, respectively. The different training/test combinations used are described in Table 1. Note that, for the combinations of train HDR/test SGE and train SGE/test HDR, the endophenotypes used for training and those providing the testing sets, i.e., the observations, corresponded to different assays. The dashed lines indicate the functionality thresholds defined in the literature for these assays and used in this work to binarize the prediction output into benign/pathogenic classes.

For BRCA1 (Figure 4), we showed the results for the MLR, Elastic and Kernel regressions (results for Ridge and Lasso are shown in Supplementary Figure S1). As we can see, the *p* values indicated a moderate, but significant, predictive ability for the different regressions. Most of the results for the train SGE/test SGE and train HDR/test SGE combinations were comparable (Figure 4a–e), with the exception of the Kernel regression applied to the train HDR/test SGE (Figure 4f). In this case, the resulting *p* value = 0.19, was lower than the other cases of the same train/test combination (*p* = 0.43). A visual inspection of the corresponding plot confirmed a poorer predictive power for this model.

The train SGE/test HDR cases deserve special mention, because their *p* values were the highest (in the vicinity of 0.6). This improvement may be due to the fact that the training set was the largest (1800 variants), and the parameter estimates were, consequently, more accurate. However, we cannot discard the possibility of a small-sample effect, resulting from the reduced number of variants, 44, in the test set.

**Figure 5:**
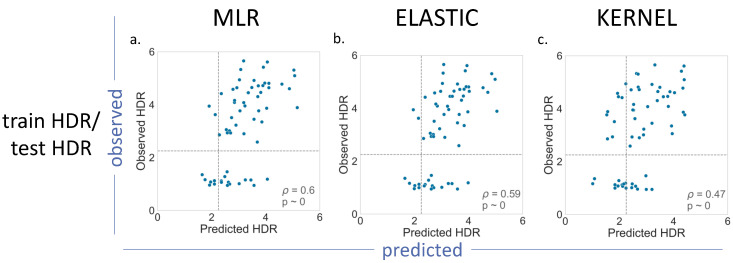
Observed vs. Predicted endophenotypes for the (**a**) MLR, (**b**) Elastic and (**c**) Kernel regressions. BRCA2 case. Here, the number of training/test combinations is lower than for BRCA1. The training and test sets correspond (see Materials and Methods) to 75% and 25%, respectively, of the HDR variant dataset for this protein. The dashed lines indicate the functionality thresholds defined in the literature for these assays and used in this work to binarize the prediction output into benign/pathogenic classes.

For BRCA2 (Figure 5), we had a smaller number of (i) variants with functional assays available and (ii) less possible train–test combinations (Table 1). The results obtained for MLR, Elastic and Kernel (Figure 5), and for Ridge and Lasso (Supplementary Figure S2), were not substantially different from those found for BRCA1: *p* values in the moderate range (0.47–0.60), with Kernel regression showing the poorest results (*p* = 0.47).

An aspect worth noting for both proteins is that all the parametric models (MLR, Ridge, Lasso and Elastic) displayed low or very low coefficients for the van der Waals volume and hydrophobicity features, the two new descriptors of the amino acid changes. In fact, the Lasso and Elastic methods favored an explicit elimination of hydrophobicity (Supplementary Table S1).

In summary, we found that, for BRCA1/2, and within the limits imposed by our datasets, we could estimate the change of an endophenotype upon a mutation with moderate accuracy.

#### 2.1.3. The Effect of the MSA on Endophenotype Estimates

All the results in the previous sections were reproduced using Hmsa instead of Omsa (Supplementary Figures S3–S5). We observed some minor differences in the *p* values relative to the Omsa results; for example, the Elastic model for the pair train SGE/test SGE gave 0.49 and 0.47 for Omsa and Hmsa, respectively, the Kernel approach for the pair train SGE/test HDR gave 0.54 and 0.60, respectively, etc.

Overall, the results obtained supported, within the limits of our data, our previous conclusion: for BRCA1 and BRCA2, we can estimate, with moderate accuracy, the endophenotype change upon mutation.

### 2.2. Are Endophenotype Estimates Good Enough for Pathogenicity Prediction?

In this section, we explore how good endophenotype estimates are for pathogenicity predictions, i.e., to which extent we can predict if a variant is pathogenic or benign using the results of the regression models. To test this idea, we followed a four-step procedure. First, we built a set of variants of a known pathogenic nature, which we referred to as the CP dataset (see Material and Methods). Second, we retrained the regression models, excluding the CP variants, from the regression training set. Third, we applied the new models to the CP variants. Fourth, we transformed the resulting outputs into binary (pathogenic/benign) outputs using the functionality thresholds given in the literature (see Materials and Methods). Here, we compared the resulting predictions with the known pathogenic nature of the variants using a series of performance measures [27,28].

**Figure 6:**
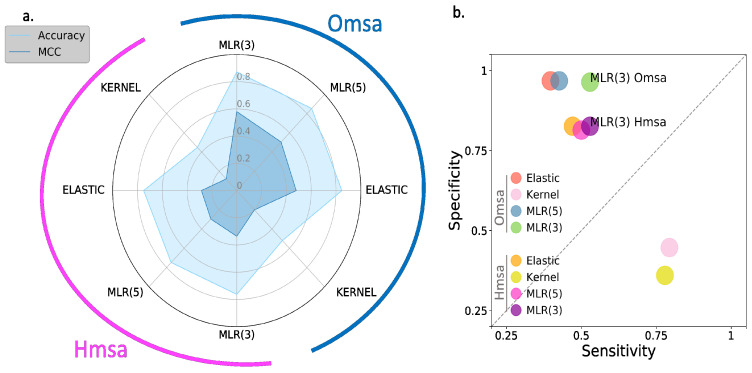
Performances of endophenotype-based pathogenicity predictions. BRCA1 case. Using functionality thresholds (see Material and Methods), we transformed the endophenotype estimates into benign/pathogenic predictions. The success rate of these pathogenicity predictions is described using four standard parameters: (**a**) Accuracy/MCC and (**b**) sensitivity/specificity. In (**a**), the half-circles external to the radar plot identify the results obtained with different multiple sequence alignments: Omsa (blue) and Hmsa (pink). In (**b**), we use different colors to establish this difference. For reference, we locate the MLR model obtained using three features only.

**Figure 7:**
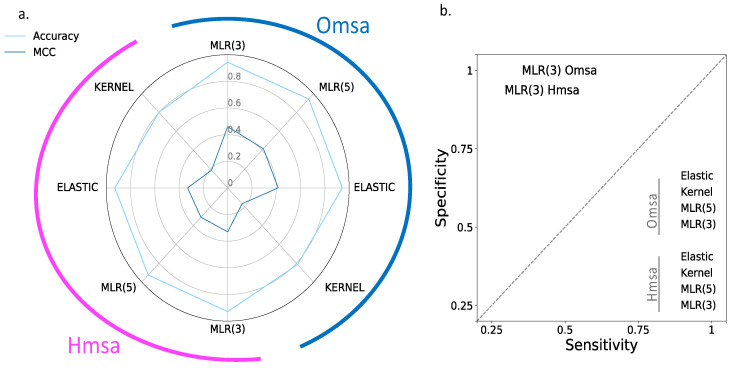
Performance of endophenotype-based pathogenicity predictions. BRCA2 case. Using functionality thresholds (see Material and Methods), we transformed the endophenotype estimates into benign/pathogenic predictions. The success rate of these pathogenicity predictions is described using four standard parameters: (**a**) Accuracy/MCC and (**b**) sensitivity/specificity. In (**a**), the half-circles external to the radar plot identify the results obtained with different multiple sequence alignments: Omsa (blue) and Hmsa (pink). In (**b**), we use different colors to establish this difference. For reference, we located the MLR model obtained using three features only.

In Figure 6 and Figure 7, we show the results obtained for BRCA1 and BRCA2, respectively, focusing on four descriptors (Supplementary Table S2): Matthew’s correlation coefficient (MCC), accuracy, sensitivity and specificity (see Material and Methods). We see the following general trends, common to both proteins.

First, the Kernel-based predictions had the poorest performances of all. We excluded them from the next observations.

Second, there was an apparent contradiction between the moderately good values for accuracy (75–87% for BRCA1; 80–94% for BRCA2) and the more modest MCC values (0.34–0.58 for BRCA1; 0.2–0.4, for BRCA2). This contradiction is explained by the considerable imbalance between specificity and sensitivity for all the methods (Figure 6b and Figure 7b for BRCA1 and BRCA2, respectively); while the specificity is over 80% for both proteins, the sensitivity is closer to, or below, 50% for BRCA1 and clearly below 50% for BRCA2.

Third, the imbalance between specificity and sensitivity is higher for BRCA2 (Figure 7b) than for BRCA1 (Figure 6b) and is consistent with the lower MCCs displayed for BRCA2.

Fourth, the predictive performance of the Omsa-based methods was better than that of the Hmsa-based methods (Supplementary Table S2), particularly if we focused on the MCC. However, in both cases, the MCCs were higher than zero, indicating a discriminant ability different from that of a random method.

In summary, the computational endophenotype estimates can be transformed into pathogenicity predictions, with moderate success rates, although with a bias towards higher specificities. Overall, the performance was better for BRCA1 than for BRCA2.

### 2.3. Benchmarking Endophenotype-Based Pathogenicity Predictions against Standard In Silico Predictions

In this section, we explore if the endophenotype-based pathogenicity predictions from the previous section are comparable to those generated by standard pathogenicity predictors. The latter, routinely used in the clinical setting [15], treat the pathogenicity prediction problem as a binary classification problem in which variants can be either pathogenic or benign and which are solved using any of the multiple machine learning tools available. We used two representatives of the standard pathogenicity prediction approach: (i) a set of ten known in silico tools (see Material and Methods) and (ii) two in-house predictors developed for this analysis: Random Forest (RF) and a Neural Network (NN) (see Material and Methods).

We developed the RF and NN predictors to allow a fairer comparison between the endophenotype-based predictors and standard pathogenicity predictors, because standard tools, like PolyPhen-2, REVEL, etc., use discriminant features other than ours and are trained with multigene variant sets. We built our RF and NN predictors with the same five features used to develop the endophenotype regressions and employed the CP dataset for training/test purposes, following a standard LOOCV procedure.

The performances of the different methods were described using several parameters (see Material and Methods); however, for simplicity, we focused our analysis on the MCC, a parameter that gives a balanced view of the performances of in silico tools and is commonly utilized in the field [17].

**Figure 8:**
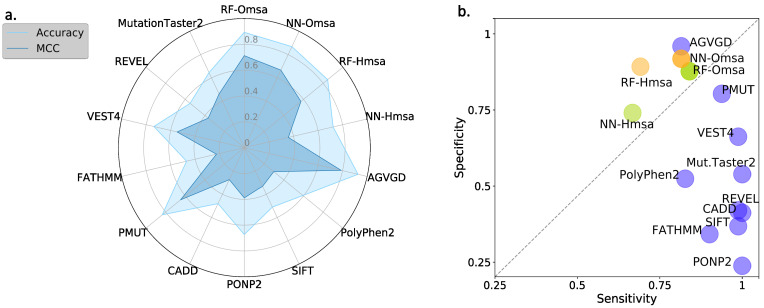
Performances of the standard pathogenicity predictors for the BRCA1 variants, described using four standard parameters: (**a**) Accuracy/MCC, and (**b**) sensitivity/specificity. Here, we compare the performances of ten well-known pathogenicity predictors with that of a family of RF and NN predictors for BRCA1. The RF/NN predictors were derived for this study using the five properties employed to characterize the variants and a set of BRCA1 variants with known pathogenicity annotations (see Materials and Methods). We added Hmsa and Omsa to their names to indicate the origin of the msa utilized to compute the conservation-based properties.

**Figure 9:**
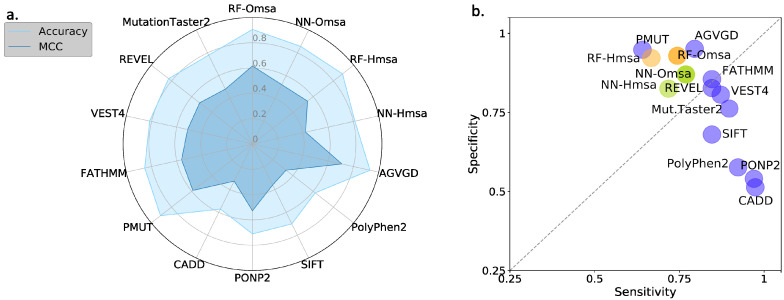
Performances of the standard pathogenicity predictors for the BRCA2 variants, described using four standard parameters: (**a**) Accuracy/MCC, and (**b**) sensitivity/specificity. Here, we compare the performances of ten well-known pathogenicity predictors with that of a family of RF and NN predictors for BRCA2. The RF/NN predictors were derived for this study using the five properties employed to characterize the variants and a set of BRCA2 variants with known pathogenicity annotations (see Materials and Methods). We added Hmsa and Omsa to their names to indicate the origin of the msa utilized to compute the conservation-based properties.

In Figure 8 and Figure 9, we compare the performances of the ten selected pathogenicity predictors and those of the RF and NN to show that the latter are good representatives of the standard approach. We describe below the main trends observed that are common to BRCA1 and BRCA2.

First, the performances of RF and NN are similar, although from the point of view of MCC, particularly when using Omsa, RF have a somewhat better performance. For this reason, and to favor presentation clarity, in the following, we will restrict our analysis to RF predictors.

Second, RF predictors are competitive, with MCCs (BRCA1:0.71-BRCA2:0.62) comparable or better than those of the standard tools (Supplementary Table S3) like PMut (BRCA1:0.65-BRCA2:0.59), VEST4 (BRCA1:0.55-BRCA2:0.51), REVEL (BRCA1:0.37-BRCA2:0.52), etc. In fact, only Align-GVGD (BRCA1:0.79-BRCA2:0.71) has better MCCs than the RFs. For BRCA1, the balance between the sensitivity and specificity values (Figure 8b) for RF-Omsa was better than that of any other tool. For BRCA2, the situation was somewhat different, some of the best-performing standard tools (FATHMM, REVEL and VEST4) showing better balances than RF-Omsa. The competitiveness of RFs dropped a little when using Hmsa instead of Omsa (Figure 8 and Figure 9); however, they still had a significant success rate.

**Figure 10:**
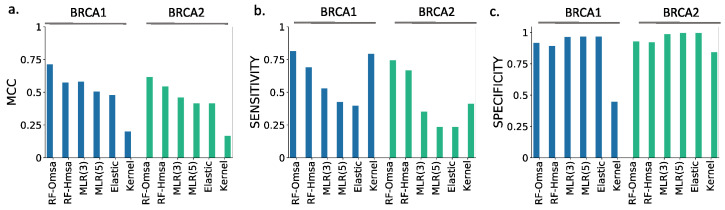
Performance comparisons between the RF and endophenotype-based pathogenicity predictions. This figure shows the values of three key descriptors of the success rate: (**a**) MCC, (**b**) sensitivity and (**c**) specificity. The results for BRCA1 and BRCA2 are colored in blue and green, respectively.

In summary, these results confirmed that RFs trained with the features used in this work have a good success rate in the pathogenicity prediction of BRCA1/2 missense variants, comparable to that of the standard tools. If we now compare the performance of the RF with that of the endophenotype-based pathogenicity predictions, we find that the former outperforms the latter for MCC (Figure 10a) and sensitivity (Figure 10b). For specificity (Figure 10c), this trend is somewhat reversed, but the differences are minor.

When comparing the endophenotype-based pathogenicity predictions (Figure 6 and Supplementary Table S2) with those from the known pathogenicity predictors (Figure 8 and Supplementary Table S3), like PolyPhen-2, PON-P2, REVEL, etc., we found that the MLR models had better MCCs for BRCA1 (except for Pmut and Align-GVGD), a trend reversed for BRCA2. It is interesting to note that, for both proteins, endophenotype-based pathogenicity predictions have specificities clearly above sensitivities, while the opposite is the case for the standard pathogenicity predictors, with the exception of Align-GVGD and Pmut (for BRCA1).

**Figure 11:**
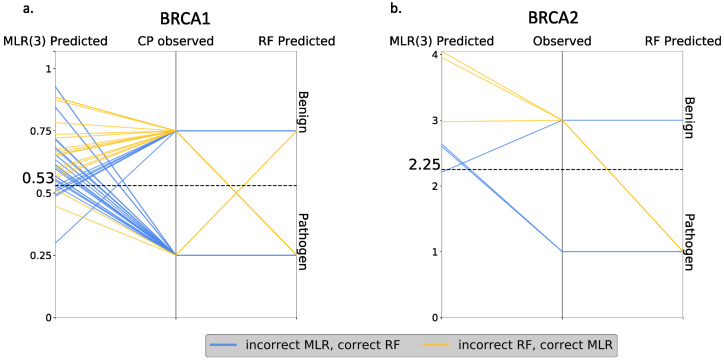
Variant-level description of the discrepancies between the endophenotype-based pathogenicity predictions and RF. In (**a**,**b**), we have the same plot for BRCA1 and BRCA2, respectively. The three vertical lines in each plot represent, from left to right, the following: the values of the endophenotype estimates (obtained with MLR), the actual pathogenic natures of the variants (benign-top/pathogenic-bottom) and the RF predictions for the variants. In this representation system, each variant is characterized by a triplet, e.g., (0.75, Benign and Pathogen); these values are united by a line, which, in our example, would go from 0.75 (first axis) to Benign (second axis) to Pathogen (third axis). We display only the lines for the variants for which MLR was successful and RF failed (yellow) and vice versa (blue). The dashed line indicates the functionality threshold for the HDR assay.

Finally, we compared RF and MLR (the best regression model) at the variant level, looking for discrepancies between both approaches. The idea was to establish how RF outperforms MLR: (i) by correctly predicting the same variants as MLR, plus some new cases, or (ii) by increasing the overall number of correct predictions, regardless of whether they coincide or not with the correct MLR predictions. In the first case, one method represented an absolute advance over the other, while, in the second case, there was a certain complementarity between them. As shown in Figure 11, we were in the second situation, i.e., RF failed the predictions for some variants that were correctly predicted with MLR. Interestingly, we also saw that part of the RF failures were borderline cases from the endophenotype point of view.

In summary, the performances of the endophenotype estimates when used for pathogenicity prediction were comparable to that of the standard tools in the field in the case of BRCA1 and somewhat lower for BRCA2. It was below that of a RF tool trained with the same features, but these tools showed a complementary behavior.

## 3. Discussion

Presently, there are tenths of in silico tools for a pathogenicity prediction, most of which are easily accessible to interested users [15]. They are normally trained using sets of pathogenic and benign variants available from the literature and usually produce a binary output, which is a prediction of the variant’s pathogenicity [17]. Some of these tools were developed with BRCA1/2 missense variants in mind, like the well-known Align-GVGD program [10]. An overview of the state-of-the-art developments in this specific field and, more precisely, of the performances of the known tools in the BRCA1/2 variants can be found in the Human Mutation issue dedicated to the CAGI 5 challenge [16]. There, Cline et al. [23] reviewed the results for the ENIGMA section (devoted to BRCA1/2), concluding that, while the computational methods show positive features, they are not yet ready for stand-alone usage. Here, we explored one of the strategies presented in the CAGI 5 challenge [22] developed by our group and based on the prediction of functional assays, also referred to as endophenotypes [19].

The idea of using endophenotypes for pathogenicity predictions was discussed by Masica and Karlin [19] in an article that provided a clarifying view on the prediction problem. Endophenotypes are quantitative measurements, like the results of the functional assays used for the BRCA1/2 variants, that can be easier to predict because of their proximity to the genotype [19]. This proximity may simplify predict the model, by reducing the need for terms representing a genetic background. This is an important advantage because of the high complexity of these terms [29] and the lack of data for their appropriate representation. Recently, we developed an implementation of the endophenotype approach, specific for the BRCA1/2 variants, fitting a multiple linear regression, MLR, to the results of the HDR assays available in the literature [22]. When presented to CAGI 5, the MLR predictions were competitive relative to those from the other approaches standard or specific for the challenge [22,23]. In fact, if we consider the best results submitted by each team, our technology ranked second for all the performance measures considered [23]. The top-ranking methodology was LEAP [23], a tool presented by the Color Genomics company that integrates heterogeneous information, including population frequencies; predictions from several in silico tools (PolyPhen-2, SIFT, etc.), information from the literature; etc. While the performance of this method was undeniably good; the fact that, in some cases, literature reports were key to its success may reduce its applicability for rare variants [30]. Additionally, the heterogeneity of the input features, apart from hindering the post hoc interpretation of the results, may pose problems if we want to add, in a coherent way, additional terms required to represent a genetic background and make the predictions more patient-specific.

In this context, we believe that our results at CAGI 5 are consistent with the idea that endophenotypes can be competitively used for a pathogenicity prediction. However, because of the nature of the challenge, systematic tests to delimit the reach of the approach could not be done. Here, we address this issue, exploring the impact on the performance of (i) a better representation of the amino acid change (using two new amino acid properties) and (ii) a different msa to compute the conservation properties. For the former, we included, in the initial regression model, two new variables previously tested for the prediction of the clinical phenotype [22,31]: volume and hydrophobicity changes upon the mutation, respectively. We then explored if the resulting increase in model complexity (from three to five variables) was penalized by the regularization techniques (Ridge, Lasso and Elastic regressions) aimed at reducing the model complexity. The results obtained (Figure 4 and Figure S1) for BRCA1 and (Figure 5 and Figure S2) for BRCA2 showed that, for the nonregularized MLR, the coefficients of the two new variables were almost negligible (Supplementary Table S1). This situation does not change by the application of the regularization techniques, who either favors zero (Lasso and Elastic) or very small values (Ridge) of the corresponding regression coefficients. In summary, the proposed refinement in the amino acid representation did not significantly improve our ability to predict the endophenotype based on the original three properties.

Subsequently, we tested the suitability of endophenotype estimates for the pathogenicity prediction. To this end, after binarizing the outputs [22] of the regression models, we tested their performances in the prediction of the pathogenic class (benign or pathogenic) of the variants. The results indicated that all the models had a certain predictive power (Figure 6 and Figure 7) and that, among them, MLR was the one with the highest MCC values. However, all the predictors, including MLR, presented an unwanted imbalance between specificity and sensitivity. This situation happens for both BRCA1 and BRCA2, and its origin is unclear. We believe that it can be partly attributed to another, more fundamental, imbalance at the sample level, where variants are distributed unequally across the functional range of the assay. For example, for the SGE assay [21], there are more nondisruptive than disruptive variants, and this compositional difference may bias the training of the regression models [32]. To explore whether this was the case, we reproduced all the results in Figure 4, Figure 5, Figure 6 and Figure 7, introducing a resampling step [32,33] in our original training procedure (Figure 2). The resampling generated a new training set with even more a presence of function disruptive and nondisruptive variants. The results obtained with the new training set are presented in Supplementary Figures S6 and S7. First, we saw that, although endophenotypes are reproduced more or less similarly, the new estimates (blue dots) showed an identifiable shift for the points corresponding to the disruptive cases. This difference translated, when moving to the pathogenicity prediction results, into more balanced sensitivity/specificity values and, clearly, better MCCs (Supplementary Figures S8–S10). This result indicated that a sample composition/structure has to be taken into account when building endophenotype predictors.

In this context, we may wonder whether transforming endophenotype predictions into pathogenicity predictions is preferable to using the standard pathogenicity predictions. Our results at CAGI 5 showed that, for BRCA1/2, endophenotype-based predictions can outperform many conventional in silico tools. Here, to better compare the two approaches, we built a Random Forest predictor using the same properties employed for the regression models but trained to predict the binary pathogenic/benign nature of the variants. We found that this RF outperformed essentially all the standard methods tried (REVEL, PolyPhen-2, etc.), with the exception of Align-GVGD, which can be explained by the fact that Align-GVGD is normally used for the annotation of BRCA1/2 variants [13]. We see that the RF also outperformed the endophenotype-based predictions (Figure 10). On this basis, we may conclude that it is preferable to address a pathogenicity prediction using the standard approach rather than endophenotype-based tools. However, a comparison at the variant level of the two approaches (Figure 11) showed that, for some cases, the endophenotype-based predictions were correct while the corresponding RF predictions failed. This indicated the existence of a certain degree of complementarity between both approaches that supported their combined use. In addition, the utilization of endophenotype estimates for a pathogenicity prediction is still in its infancy, and simple technical improvements, like a careful choice of the msa (e.g., Omsa instead of Hmsa) or resampling of the regression training set, may result in substantial performance improvements. This is particularly true in the case of resampling, which results in regression models leading to better pathogenicity prediction success rates (Supplementary Figures S8–S10). In fact, the results obtained point to success rates superior to those of the standard methods (including Align-GVGD) for BRCA1 (comparing Figure 8, Figure S8 and Figure S9) and notable improvements for BRCA2 (comparing Figure 9 and Figure S10). Independently from the predictive ability, other advantages that support the use of endophenotype-based predictions were related to the interpretability of the results. In the case of regressions, the output was directly understandable in functional terms, in contrast with the output of the RF or of the other standard predictors. In addition, the use of additive models in the regressions favored a direct understanding of the predictions in terms of the input features. This degree of interpretability was not easy to reach in the RF models or for other machine learning-based tools, given their black box nature [15].

In accordance with a previous work on protein-specific pathogenicity predictors [34], we found that the success rate of our endophenotype-based pathogenicity predictions was different between BRCA1 and BRCA2, being lower for the latter. This difference, already noted by Hart et al. [35] in their BRCA1/2 pathogenicity predictors, indicated that a genetic background may not be the only source of variability in the performances of the in silico tools. The properties related to the molecular functions of the protein, like 3D structure or pattern of protein–protein interactions, etc., are different between BRCA1 and BRCA2 and may not be equally captured by the msa-based properties included in our models. This suggests that the estimates of the BRCA2-related endophenotypes may require the use of additional properties.

In summary, our results confirmed that it is possible to computationally estimate the endophenotype change upon mutation, i.e., the output of functional assays, for BRCA1/2 proteins. These estimations may reach an accuracy level compatible with a subsequent use for pathogenicity predictions, although in conjunction with other in silico tools. We found that the training of the regression models had some technical subtleties, like the adequate use of msa or the application of resampling techniques. However, in exchange for these minor difficulties, the endophenotype-based predictions had the advantage of their good interpretability. We hope that future efforts will increase their success rates, bringing them closer to clinical applications.

## 4. Materials and Methods

### 4.1. The Variant Datasets

In Figure 2, we show the training scheme used to develop our computational estimates of the endophenotype. The first requirement of this procedure is the availability of missense variant data with known endophenotypes. That is, datasets of variants for which the impact on the protein function was measured using an experimental assay. The outcome of the chosen assay will constitute our target endophenotype, i.e., our modeling goal. Here, we used two such datasets (Figure 1), HDR and SGE, that we describe below.

HDR. These are two datasets, one for BRCA1 and the other for BRCA2, of variants for which we know the outcome of the HDR functional assay. This assay is used to measure the change upon mutation in the repair of DNA double-stranded breaks by the homology-directed repair mechanism that depends on these proteins. We retrieved published values of the assay for BRCA1 [24] and BRCA2 [25] missense variants. The final sets were constituted by 44 variants (17 pathogenic and 27 benign) for BRCA1 and 252 variants (90 pathogenic and 162 benign) for BRCA2.

SGE. This dataset was obtained from the work by Findlay et al. [21], where 96.5% of all possible single-nucleotide variants of BRCA1 in exon 13 were functionally scored with saturation genome editing. A total of 1837 missense variants were retrieved from the paper. We used the functional classification of the authors [21] to label the variants as pathogenic (score < −1.328), benign (score > −0.748) and intermediate (−1.328 < score < −0.748). The final set was constituted by 393 pathogenic, 1276 benign and 168 intermediate variants. These numbers varied slightly depending on the training scheme followed to develop our computational models (Table 1).

We used a third variant dataset, CP (which stands for Clinical Phenotype), constituted by variants with a known, binary clinical phenotype (pathogenic/benign). It is the result of merging, independently for BRCA1 and BRCA2, two datasets: (i) a manually curated set of missense variants used for the initial training of our pathogenicity predictors [22] and (ii) the set of missense variants proposed in the CAGI 5-ENIGMA challenge [23,36] for BRCA1/2. For the variants in the CAGI 5 set, the IARC-5 tier classes were converted to binary classes. The resulting CP dataset consisted of 350 variants (81 pathogenic and 269 benign) for BRCA1 and 308 variants (39 pathogenic and 269 benign) for BRCA2. We used CP for testing how good are endophenotype-based pathogenicity predictions were compared with those of the standard in silico tools (like REVEL, PON-P2, PolyPhen-2, etc.).

These datasets were used in different training/testing set combinations to characterize the performances of our computational tools. These combinations of datasets and the predictors for which they were used are described in Table 1.

### 4.2. The Predictive Features

We used five features in our models. These features corresponded to function-related properties of the variants; we used them in previous pathogenicity prediction studies [34,37], with good results. We briefly describe them here, but more information can be found in references [31,34,37]. Two of the features are conservation descriptors derived from the multiple sequence alignment (msa) of the target protein’s family: (i) Shannon’s Entropy, which is equal to:

(1) $$\sum_{i}p_{i}\;\cdot \;\log\left(p_{i}\right)$$

where $p_{i}$ is the frequency of an amino acid with index i at the variant’s location in the msa and (ii) Position-specific scoring matrix element for the native amino acid, which is equal to:

(2) $${\log(f}_{\mathrm{nat},i}/f_{\mathrm{nat},\mathrm{MSA}})$$

where $f_{\mathrm{nat},i}$ is the frequency of the native amino acid at the variant’s location in the msa, and $f_{\mathrm{nat},\mathrm{MSA}}$ is the frequency of the same amino acid in the whole msa. These two features were computed independently for two different msa. One was retrieved from the Align-GVGD server (http://agvgd.hci.utah.edu, accessed on 5 March 2018), where the manually curated msa of BRCA1/2 orthologs are freely available from the authors of this prediction software. We will refer to these msa as Omsa. The second set of msa, constituted by homologs of BRCA1/2, were built following the automatic procedure described in Riera et al. [34]. We will refer to these msa as Hmsa.

The other three features are properties that reflect the intrinsic nature of the amino acid replacement [34] independently of its location in the protein: the elements of the Blosum62 mutation matrix and two indexes accounting for the differences between the native and the mutant residues in the van der Waals volume and hydrophobic character, respectively.

Note that Shannon’s entropy, position-specific scoring matrix and Blosum62 were used to build the MLR model we presented at the CAGI 5 challenge [22].

### 4.3. Regression Models

To explore how well we can estimate the endophenotype change upon mutation, which is a continuous value, we tested five regression models (Multiple Linear Regression or MLR, Ridge, Lasso, ElasticNet and KernelRidge). All of them are available from the python package Scikit-learn [38]. All the models, except MLR, were subjected to a hyperparameter tuning process with a cross-validation, following the scheme in Figure 2.

We tested the regression models for the following training/test combinations. For BRCA1, we used train SGE/test SGE, in which the SGE dataset was split (75% training and 25% testing), train HDR/test SGE, in which we used the HDR and SGE datasets for training and testing, respectively, and train SGE/test HDR, which corresponded to the opposite combination. For BRCA2, as only HDR data were available, we used train HDR/test HDR, in which the HDR dataset was split (75% training and 25% testing).

For MLR models, for which no hyperparameter tuning was required, in addition to the previous train/test combinations, we also tried a standard leave-one-out cross-validation [34] (LOOCV) using the HDR datasets (Figure 3a).

The remaining four regression models (Ridge, Lasso, Elastic and Kernel) required a hyperparameter tuning step, as shown in Figure 2. The hyperparameters were optimized using the GridSearchCV option; for Ridge, Lasso and ElasticNet, this meant using a LOOCV scheme, and, for KernelRidge, a three-fold cross-validation scheme. In this process, the minimum/maximum hyperparameter values and grid size varied with the dataset. For the Ridge models, the alpha hyperparameters ranged from 0.05 to 100. For Lasso, the alpha hyperparameter ranged from 0.05 to 5. For ElasticNet, the alpha values ranged from 0.05 to 5, whereas the L1 ratio ranged from 0.1 to 1. Finally, for the “rbf” KernelRidge models, the alpha hyperparameters ranged from 0.01 to 5 and gamma parameters ranged from 0.1 to 10. For all the models, to evaluate the performance on the cross-validated data during the hyperparameter tuning process, we used the “neg_mean_squared_error” scoring in the corresponding Scikit-learn [38] package. We want to note that the results of the Ridge process were almost identical to those of the MLR regression. This was due to the very low value of the optimal hyperparameter automatically selected at the end of the tuning process.

All the regression models described here were built twice, with and without applying resampling procedures to the training dataset [32,33]. For the former, we applied an under-sampling procedure using RandomUnderSampler from the Scikit-learn package. In the case of the SGE assay, the variants belonging to the intermediate class were excluded from the resampling process. The results for the non-resampled datasets are shown in the main body of the paper (MLR, Elastic and Kernel) and in the Supplementary Materials (Ridge and Lasso). The results for all five models derived from the resampled datasets are shown in the Supplementary Materials.

It is important to note that, for all the training–test combinations used in this work (Table 1), common variants were excluded from either the training or the test set to avoid overfitting problems.

Note that all the models generated were protein-specific, i.e., the BRCA1 and BRCA2 variants were not pooled together to generate the endophenotype regression models.

### 4.4. Binarization of the Endophenotype Estimates into Pathogenic/Benign Values

As part of the goal of this work, we tested how good are endophenotype estimates (the values obtained with the different regression models) were for the pathogenicity prediction using the variants in the CP dataset. To this end, we followed a two-step procedure. First, the trained regression was used to produce an endophenotype estimate for the variants in CP. Second, this estimate was binarized using functionality thresholds from the literature.

For this analysis, we used the following combinations of training/test sets. For BRCA1, we tried two options: train HDR/test CP, in which the HDR dataset was used to build the regression models and whose results were then binarized as follows: CP variants with an HDR estimated value below 0.53 [24] were labeled as pathogenic, snd those above were labeled as benign. The second option was train SGE/test CP, in which the SGE dataset was used to build the regression models, whose results were then binarized as follows: variants with an estimated SGE value below −1.328 [21] were labeled as pathogenic, and those with an estimated SGE value above −0.748 were labeled as benign, and the remaining variants were labeled as unknown and excluded from performance computations. For BRCA2, because no SGE assay was available, we only tried the train HDR/test CP combination, which was equivalent to the one described for BRCA1; here, to binarize the regression outcomes, we used a threshold of 2.25 [25].

All the endophenotype estimates and pathogenicity predictions, as well as the binarization results, for all the BRCA1/2 variants used in this work can be found in Table S4.

### 4.5. Classification Models

We employed the same input features we used for the regression models to build, independently for BRCA1 and BRCA2, two machine learning pathogenicity predictors based in the Random Forest (RF) and Neural Network (NN) algorithms, respectively. Contrary to the regression models, these two predictors are trained with a binary version (benign/pathogenic) of the variant’s clinical phenotype, not with the continuous outcome of the functional assays. These RF and NN models were built using the Scikit-learn [38] package. We employed default parameters to build the RF model, whereas, for the NN, we chose a model with one hidden layer and 5 hidden units. In addition, in both cases, the pathogenic/benign composition of the training set was balanced with SMOTE [39], as implemented in Scikit-learn [38]. The output of these tools was a binary prediction: benign or pathogenic.

We trained the RF and NN classifiers using the CP dataset (see above for a description of the dataset). The performances of the classifiers were assessed using LOOCV.

For comparison purposes, we also scored the variants in the CP dataset with ten standard pathogenicity predictors: Align-GVGD [10], SIFT [11], PolyPhen-2 [12], PONP2 [40], CADD [41] and PMut [42], whose scores were obtained from our previous study [22], and FATHMM [43], MutationTaster2 [44], REVEL [26] and VEST4 [45], whose scores were downloaded from dbNSFP [46,47].

Note that all the models generated were protein-specific, i.e., the BRCA1 and BRCA2 variants were not pooled together to generate either the RF or NN models.

### 4.6. Performance Assessment

The accuracy of the endophenotype estimates was measured using, *p*, the Spearman’s Rank correlation.

To measure the accuracy of the binary pathogenicity predictions, we utilized the following, broadly used, performance parameters [27,28]: sensitivity, specificity, accuracy, Matthews Correlation Coefficient (MCC), positive predictive value (PPV) and negative predictive value (NPV). Their formulas are given below:

Sensitivity:

(3) $$\frac{\mathrm{TP}}{\mathrm{TP}+\mathrm{FN}}$$

Specificity:

(4) $$\frac{\mathrm{TN}}{\mathrm{TN}+\mathrm{FP}}$$

Accuracy:

(5) $$\frac{\mathrm{TP}+\mathrm{TN}}{\mathrm{TP}+\mathrm{FP}+\mathrm{TN}+\mathrm{FN}}$$

MCC:

(6) $$\frac{\mathrm{TP}.\;\mathrm{TN}\;-\;\mathrm{FP}\;\cdot \;\mathrm{FN}}{\sqrt{\left(\mathrm{TP}+\mathrm{FN}\right).\;\left(\mathrm{TN}+\mathrm{FP}\right).\;\left(\mathrm{TP}+\mathrm{FP}\right)\;\cdot \;(\mathrm{TN}+\mathrm{FN})}}$$

PPV:

(7) $$\frac{\mathrm{TP}}{\mathrm{TP}+\mathrm{FP}}$$

NPV:

(8) $$\frac{\mathrm{TN}}{\mathrm{TN}+\mathrm{FN}}$$

where TP and FN are the numbers of correctly and incorrectly predicted pathogenic variants, and TN and FP are the numbers of correctly and incorrectly predicted benign variants, respectively.

## Figures and Tables

**Table 1:** Description of the training/test combinations used to develop the regression and pathogenicity prediction models in this work.

PROTEIN	TRAIN ^1^	TEST ^1^	VALIDATION ^2^	MODELS ^3^
BRCA1	HDR (44)	HDR (44)	LOOCV	MLR
	HDR (44)	SGE (1800)	Independent assays	MLR, Ridge, Lasso, Elastic, Kernel
	SGE (1800)	HDR (44)	Independent assays	MLR, Ridge, Lasso, Elastic, Kernel
	SGE (1377)	SGE (460)	Train–test split assays	MLR, Ridge, Lasso, Elastic, Kernel
	HDR (44)	CP (321)	Independent assays	MLR, Elastic, Kernel
	SGE (1686)	CP (350)	Independent assays	MLR, Elastic, Kernel
	CP (350)	CP (350)	LOOCV	Random Forest, Neural Network
BRCA2	HDR (252)	HDR (252)	LOOCV	MLR
	HDR (189)	HDR (63)	Train-test split assays	MLR, Ridge, Lasso, Elastic, Kernel
	HDR (252)	CP (252)	Independent assays	MLR, Elastic, Kernel
	CP (308)	CP (308)	LOOCV	Random Forest, Neural Network

^1^ The origin of the variants in the datasets. ^2^ Validation procedure for which the train/test combination was used. ^3^ Model type trained and validated with each train/test combination.

## Data Availability

The data presented in this study are available in the Supplementary Materials.

## Supplementary Materials

The following are available online at https://www.mdpi.com/article/10.3390/ijms22126226/s1.
