# Supplementary material for: Towards a New, Endophenotype-Based Strategy for Pathogenicity Prediction in BRCA1 and BRCA2: In Silico Modeling of the Outcome of HDR/SGE Assays for Missense Variants
Source: Int J Mol Sci. 2021 Jun 9;22(12):6226. doi: 10.3390/ijms22126226 (PMC8229251; doi:10.3390/ijms22126226)
Supplement: Supplementary file 1 [file ijms-22-06226-s001.zip › SUPP_FIGURES_TABLES/SUPP_LEGENDS.pdf]

SUPPLEMENTARY FIGURES & TABLES LEGENDS

Figure S1. Observed vs. Predicted endophenotypes for the (a,c,e) Ridge and (b,d,f) Lasso regressions. BRCA1 case. Each row corresponds to the results obtained with a specific combination of training/test datasets. For example, for "train SGE/test SGE", the SGE dataset was split into a training and a test dataset with 75% and 25% of the variants, respectively. The different training/test combinations used are described in Table 1. Note that for the combinations "train HDR/test SGE" and "train SGE/test HDR", the endophenotypes used for training and those providing the testing set, i.e. the observations, are different. The dashed lines, indicate the functionality thresholds defined in the literature for these assays and used in this work to binarize the prediction output into benign/pathogenic classes (see Results).

Figure S2. Observed vs. Predicted endophenotypes for the (a) Ridge and (b) Lasso regressions. BRCA2 case. Here the number of training/test combinations is lower than for BRCA1. Training and test sets correspond (see Materials and Methods) to 75% and 25%, respectively, of the HDR variant dataset for this protein. The dashed lines, indicate the functionality thresholds defined in the literature for these assays and used in this work to binarize the prediction output into benign/pathogenic classes (see Results).

Figure S3. Observed vs. Predicted endophenotypes for the MLR model, with conservation-based features computed using Hmsa. This figure is equivalent to Figure 3. (a) and (b) results for the regression models with three features for BRCA1 and BRCA2, respectively; (c) and (d) results for the regression models with five features for BRCA1 and BRCA2, respectively. As in Figure 3, BRCA2 results are shown only for completeness. The dashed lines, indicate the functionality thresholds defined in the literature for these assays and used in this work to binarize the prediction output into benign/pathogenic classes (see Results).

Figure S4. Observed vs. Predicted endophenotypes for the (a,f,k) MLR, (b,g,l) Ridge, (c,h,m) Lasso, (d,i,n) Elastic and (e,j,o) Kernel regressions, with conservation-based features computed using Hmsa. BRCA1 case. Each row corresponds to the results obtained with a specific combination of training/test datasets. For example, for "train SGE/test SGE", the SGE dataset was split into a training and a test dataset with 75% and 25% of the variants, respectively. The different training/test combinations used are described in Table 1. Note that for the combinations "train HDR/test SGE" and "train SGE/test HDR", the endophenotypes used for training and those providing the testing set, i.e. the observations, are different. The dashed lines, indicate the functionality thresholds defined in the literature for these assays and used in this work to binarize the prediction output into benign/pathogenic classes (see Results).

Figure S5. Observed vs. Predicted endophenotypes for the (a) MLR, (b) Ridge, (c) Lasso, (d) Elastic and (e) Kernel regressions, with conservation-based features computed using Hmsa. BRCA2 case. Here the number of training/test combinations is lower than for BRCA1. Training and test sets correspond (see Materials and Methods) to 75% and 25%, respectively, of the HDR variant dataset for this protein. The dashed lines, indicate the functionality thresholds defined in the literature for these assays and used in this work to binarize the prediction output into benign/pathogenic classes (see Results).

Figure S6. The impact of re-sampling on the regression models for the (a,d,g) MLR, (b,e,h) ELASTIC and (c,f,i) KERNEL. BRCA1 case. In each scatterplot we compare two versions of the Observed vs. Predicted relationship: (i) the original relationship (yellow) is identical to the data shown in Figure 4; (ii) the new relationship (blue), in which the Predicted values are obtained from a regression model trained after the variant dataset had undergone a re-sampling process, as explained in the Discussion.

Figure S7. The impact of re-sampling on the regression models for (a) MLR, (b) ELASTIC and (c) KERNEL. BRCA2 case. In each scatterplot we compare two versions of the Observed vs. Predicted relationship: (i) the original relationship (yellow) is identical to the data shown in Figure 5; (ii) the new relationship (blue), in which the Predicted values are obtained from a regression model trained after the variant dataset had undergone a re-sampling process, as explained in the Discussion.

Figure S8. The impact of re-sampling on the performance of endophenotype-based pathogenicity predictions described using four standard parameters: (a) Accuracy/MCC, and (b) sensitivity/specificity. BRCA1 case. This figure is equivalent to Figure 6. There are two differences between them. First, the pathogenicity predictions are no longer based in the original regression models, here they are derived from

the results of regression models obtained after a resampling of the original training dataset (see Discussion). And second, the training set here is the SGE dataset.

Figure S9. The impact of re-sampling on the performance of endophenotype-based pathogenicity predictions described using four standard parameters: (a) Accuracy/MCC, and (b) sensitivity/specificity. BRCA1 case. This figure is equivalent to Figure S8, the only difference between them is that here, the training set of the regression models was HDR instead of SGE.

Figure S10. The impact of re-sampling on the performance of endophenotype-based pathogenicity predictions described using four standard parameters: (a) Accuracy/MCC, and (b) sensitivity/specificity. BRCA2 case. This figure is equivalent to Figure S9, except that here the variants correspond to BRCA2 instead of BRCA1.

Supplementary Table S1. Regression coefficients for the different regression models in this work. Column MODEL gives the type of model trained (see Materials and Methods). Column 'TRAIN/TEST SETS' contains the abbreviation of the training/test datasets; in parentheses we show the values of the hyperparameters for the different regression models. Column INTERCEPT has the values of the intercept in each regression model. The remaining columns provide the coefficients of the different properties used in the model.

Supplementary Table S2. Performances of the endophenotype-based pathogenicity predictions. Column 'PREDICTOR' provides the name of the regression model originating the endophenotype estimates, the number of features employed (F3: three, F5: five), the msa used to compute the conservation-based properties. We only give the results for the HDR-trained regression models. The names of the remaining columns correspond to the following: MCC-Matthews Correlation Coefficient; SN-Sensitivity; SP-Specificity; ACC-Accuracy; PPV-Positive Predictive Value; NPV-Negative Predictive Value; TP-True Positives (correct pathogenic predictions); TN-True Negatives (correct benign predictions); FP- False Positives (incorrect pathogenicity predictions); FN-False Negatives (incorrect benign predictions); P-Number of pathogenic variants; N- Number of benign variants; TOTAL-Total number of variants (pathogenic+benign); COVER: fraction of variants with a prediction produced.

Supplementary Table S3. Performance comparison between standard pathogenicity predictors in the case of BRCA1/2 variants. Column PREDICTOR has the name of the tools used. All of them are mentioned (standard tools) or described (our RF and NN predictors). The names of the remaining columns correspond to the following: MCC-Matthews Correlation Coefficient; SN-Sensitivity; SP-Specificity; ACC-Accuracy; PPV-Positive Predictive Value; NPV-Negative Predictive Value; TP-True Positives (correct pathogenic predictions); TN-True Negatives (correct benign predictions); FP- False Positives (incorrect pathogenicity predictions); FN-False Negatives (incorrect benign predictions); P-Number of pathogenic variants; N-Number of benign variants; TOTAL-Total number of variants (pathogenic+benign); COVER: fraction of variants with a prediction produced.

Supplementary Table S4: Endophenotype estimates and pathogenicity predictions for all the BRCA1/2 variants used in this work. Column UNIPROT has the uniport ID of the protein (P38398 for BRCA1, P51587 for BRCA2). Column VARIANT has the variant list for which the predictions made. The names of the remaining columns correspond to all the models built in this study with the following naming structure: "uniprotID_modelName_featureNumber_msaType_trainSet_testSet". Additionally, binarized regression predictions have "_class" abbreviation, and regression models built with resampled training set have "_resampled" abbreviation in their column names.
