# Supplementary material for: Towards a New, Endophenotype-Based Strategy for Pathogenicity Prediction in BRCA1 and BRCA2: In Silico Modeling of the Outcome of HDR/SGE Assays for Missense Variants
Source: Int J Mol Sci. 2021 Jun 9;22(12):6226. doi: 10.3390/ijms22126226 (PMC8229251; doi:10.3390/ijms22126226)
Supplement: Supplementary file 1 [file ijms-22-06226-s001.zip › SUPP_FIGURES_TABLES/SUPPFIGURE6.pdf]

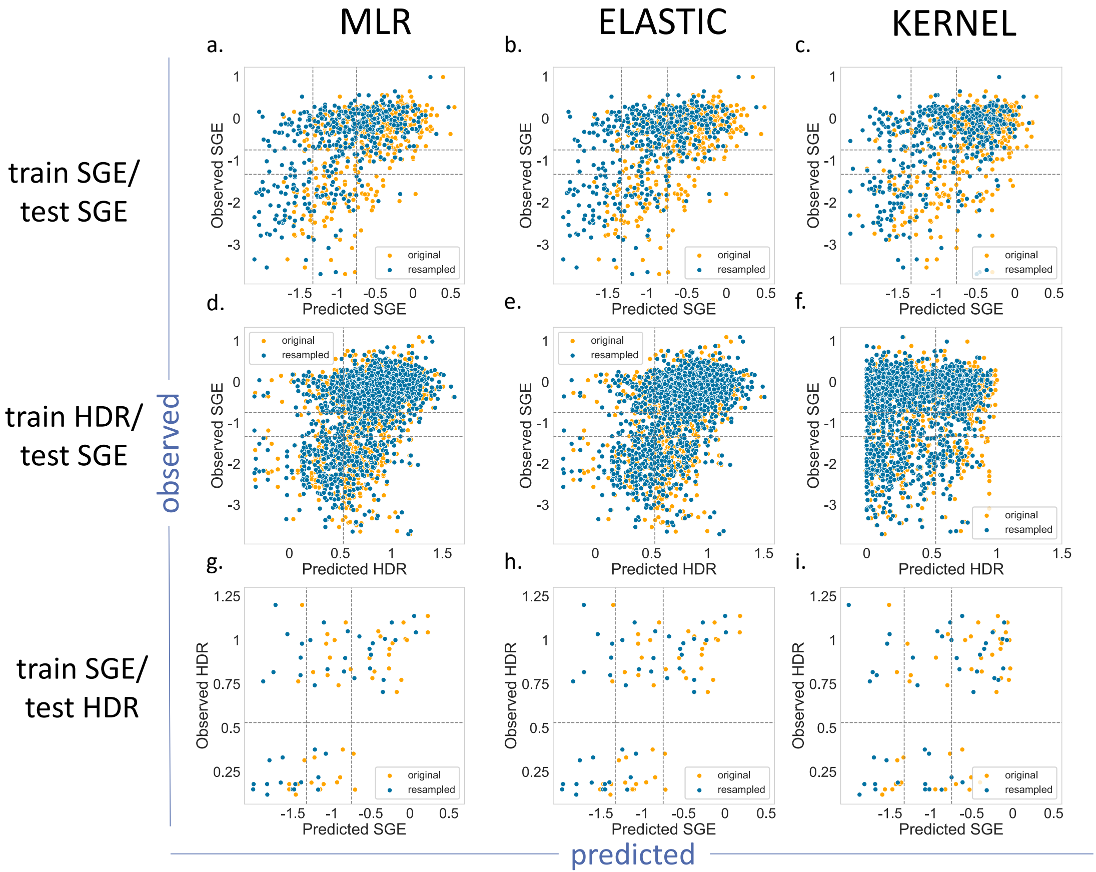

MLR
ELASTIC
KERNEL
train SGE/ test SGE
train HDR/ test SGE
train SGE/ test HDR
observed
predicted
a.
b.
c.
d.
e.
f.
g.
h.
i.
Observed SGE
Observed HDR
Predicted SGE
Predicted HDR
original
resampled
