# Supplementary figures and images for: Towards a New, Endophenotype-Based Strategy for Pathogenicity Prediction in BRCA1 and BRCA2: In Silico Modeling of the Outcome of HDR/SGE Assays for Missense Variants
Source: Int J Mol Sci. 2021 Jun 9;22(12):6226. doi: 10.3390/ijms22126226 (PMC8229251; doi:10.3390/ijms22126226)

train SGE/  
test SGE

train HDR/  
test SGE

train SGE/  
test HDR

observed

RIDGE

LASSO

a.

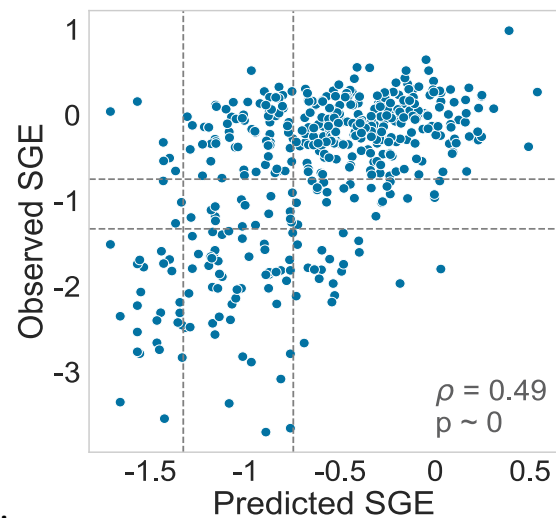

b.

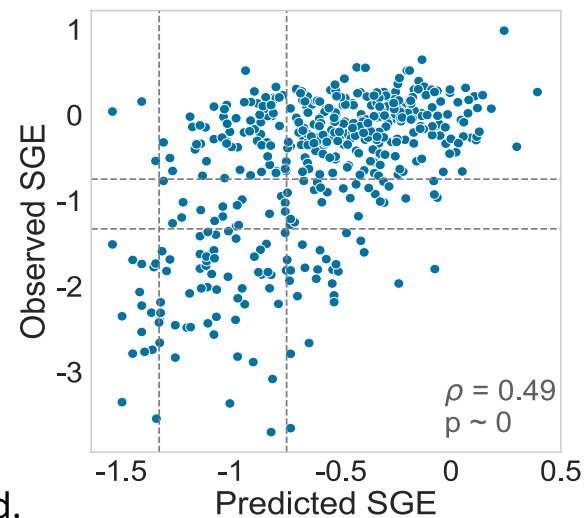

c.

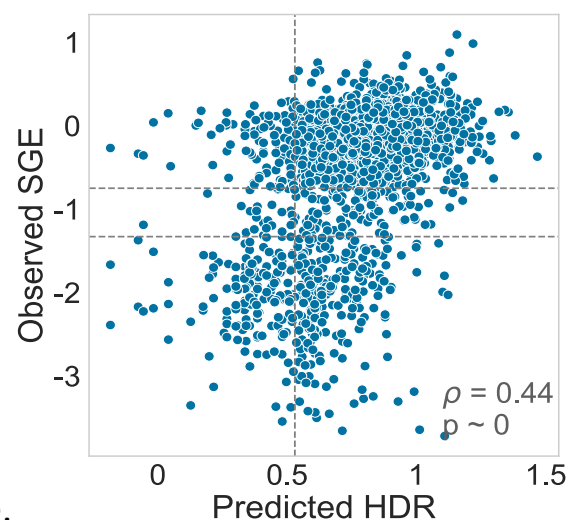

d.

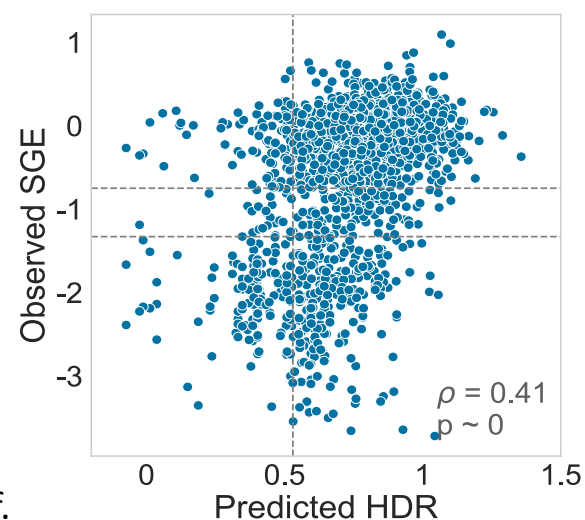

e.

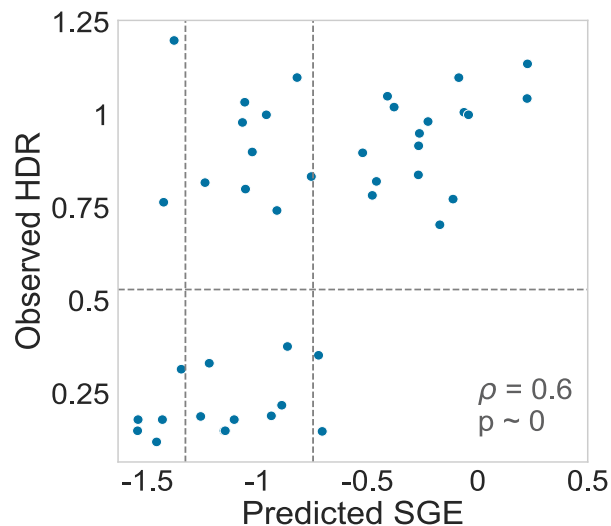

f.

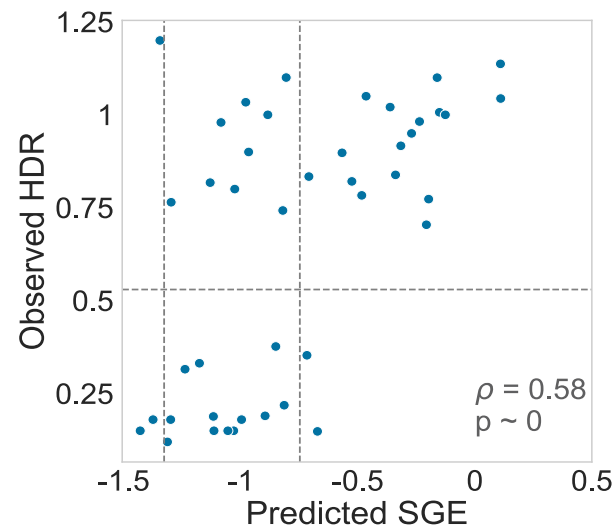

predicted

Supplement: Supplementary file 1 [file ijms-22-06226-s001.zip › SUPP_FIGURES_TABLES/SUPPFIGURE1.pdf]

a.

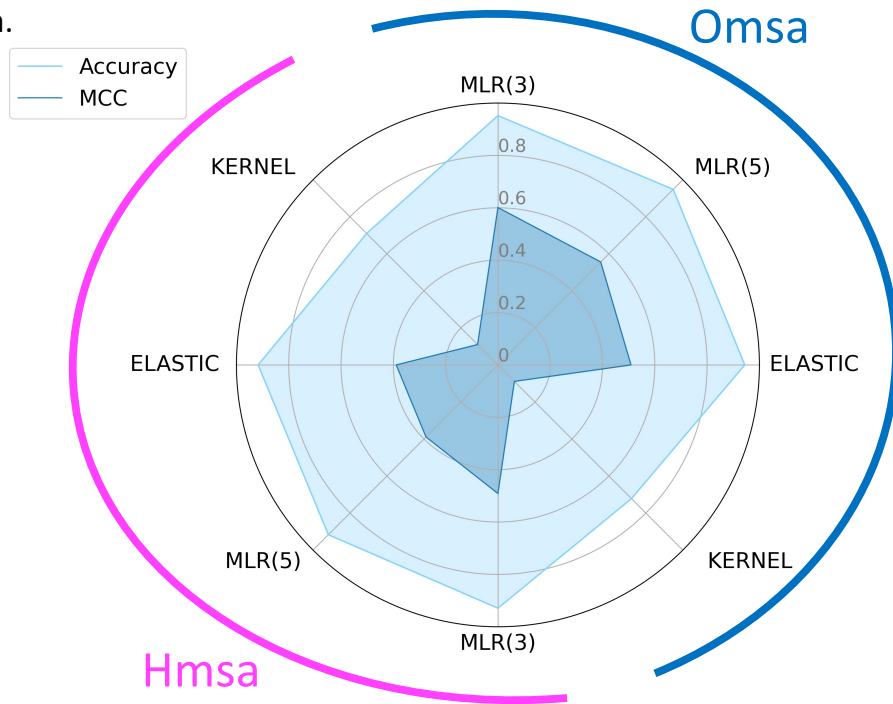

b.

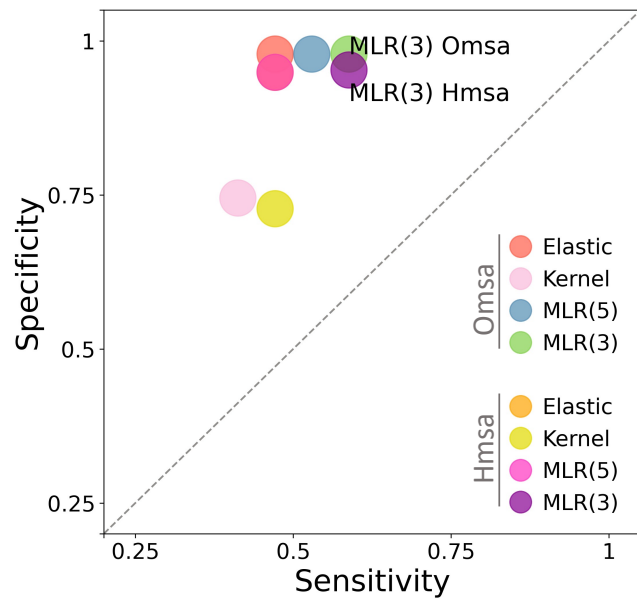

Supplement: Supplementary file 1 [file ijms-22-06226-s001.zip › SUPP_FIGURES_TABLES/SUPPFIGURE10.pdf]

train HDR/  
test HDR

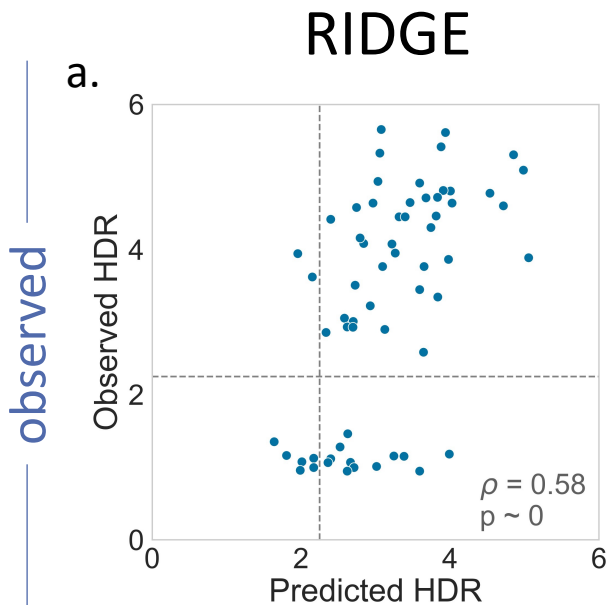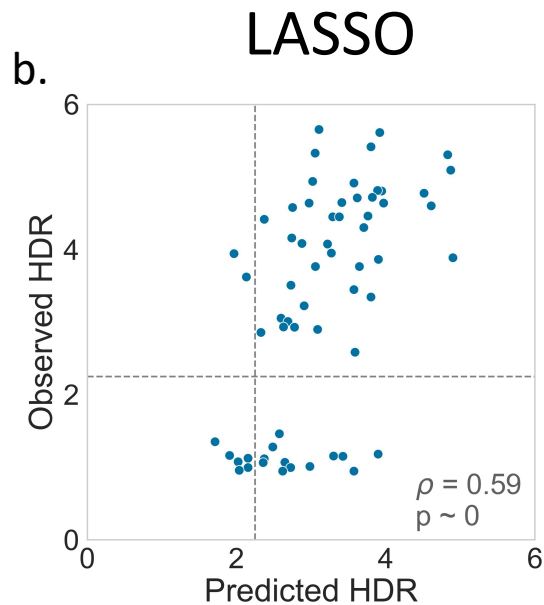

predicted

Supplement: Supplementary file 1 [file ijms-22-06226-s001.zip › SUPP_FIGURES_TABLES/SUPPFIGURE2.pdf]

## THREE FEATURES

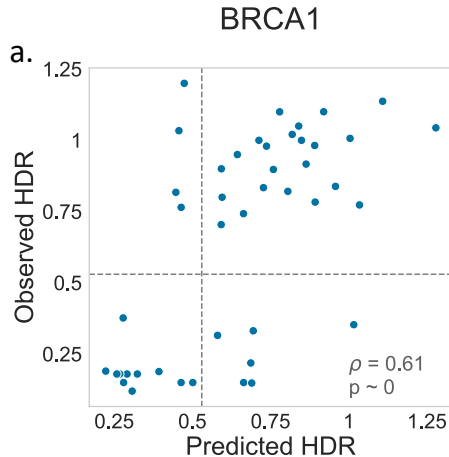

## BRCA2

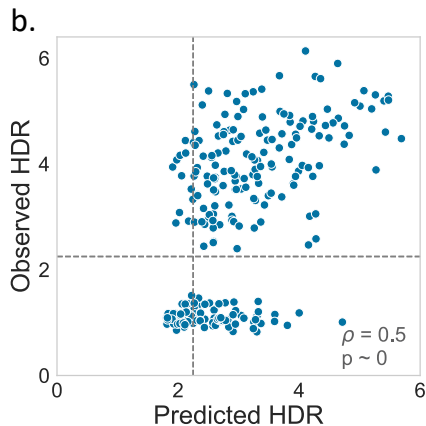

## FIVE FEATURES

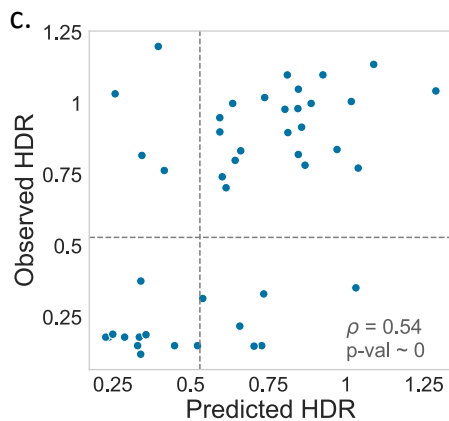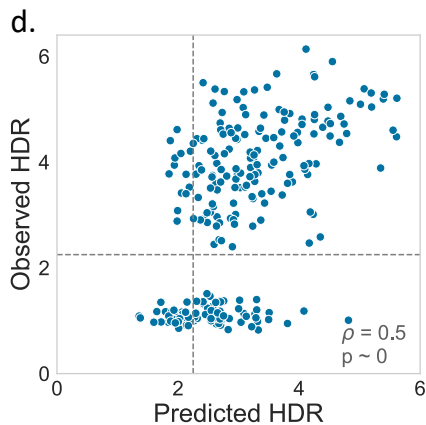

Supplement: Supplementary file 1 [file ijms-22-06226-s001.zip › SUPP_FIGURES_TABLES/SUPPFIGURE3.pdf]

train SGE/  
test SGE

train HDR/  
test SGE

train SGE/  
test HDR

observed

predicted

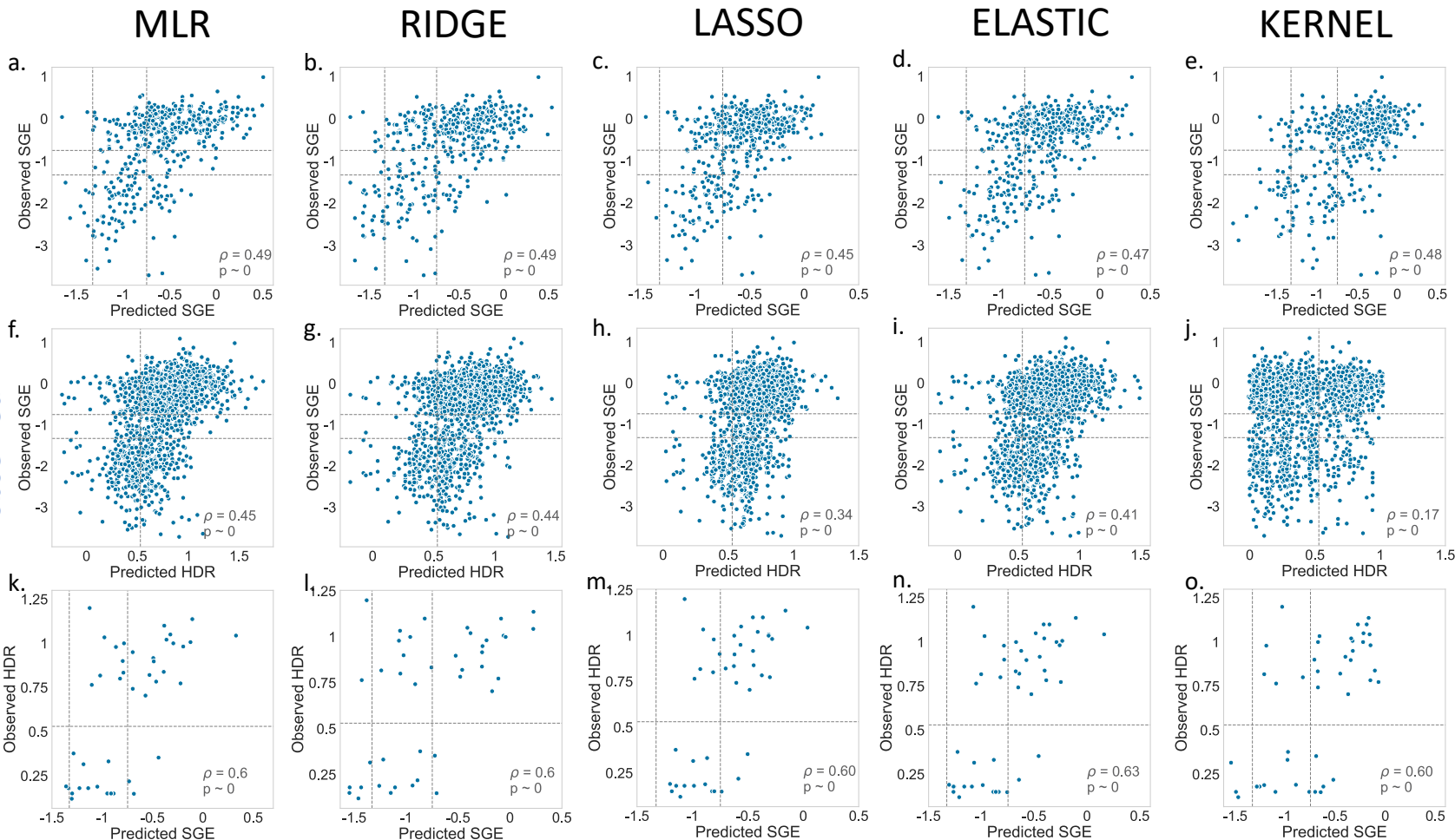

Supplement: Supplementary file 1 [file ijms-22-06226-s001.zip › SUPP_FIGURES_TABLES/SUPPFIGURE4.pdf]

train HDR/  
test HDR

observed

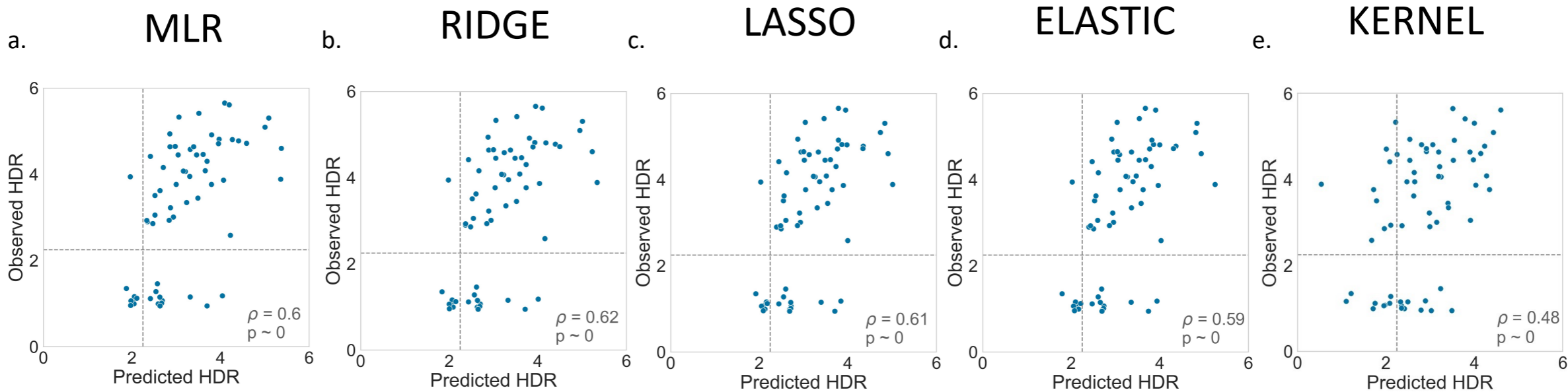

predicted

Supplement: Supplementary file 1 [file ijms-22-06226-s001.zip › SUPP_FIGURES_TABLES/SUPPFIGURE5.pdf]

train HDR/  
test HDR

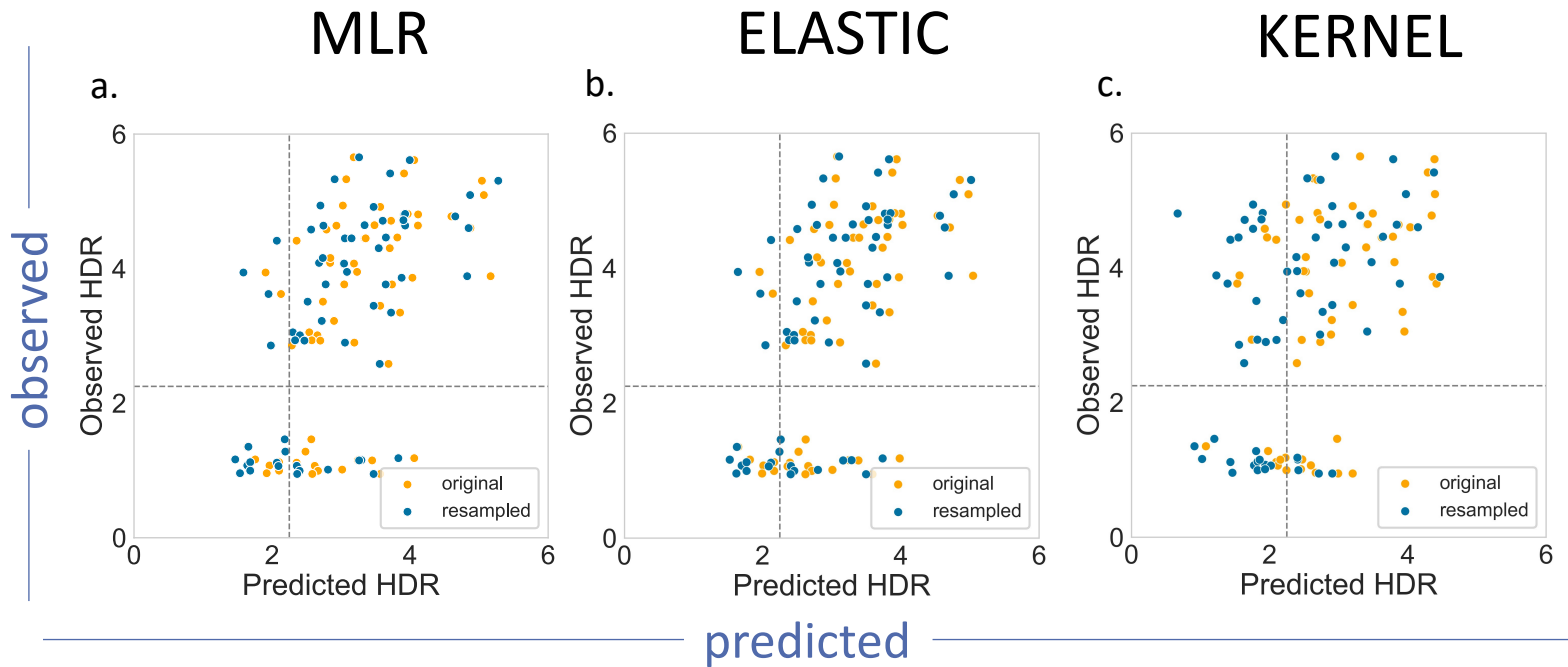

Supplement: Supplementary file 1 [file ijms-22-06226-s001.zip › SUPP_FIGURES_TABLES/SUPPFIGURE7.pdf]

a.

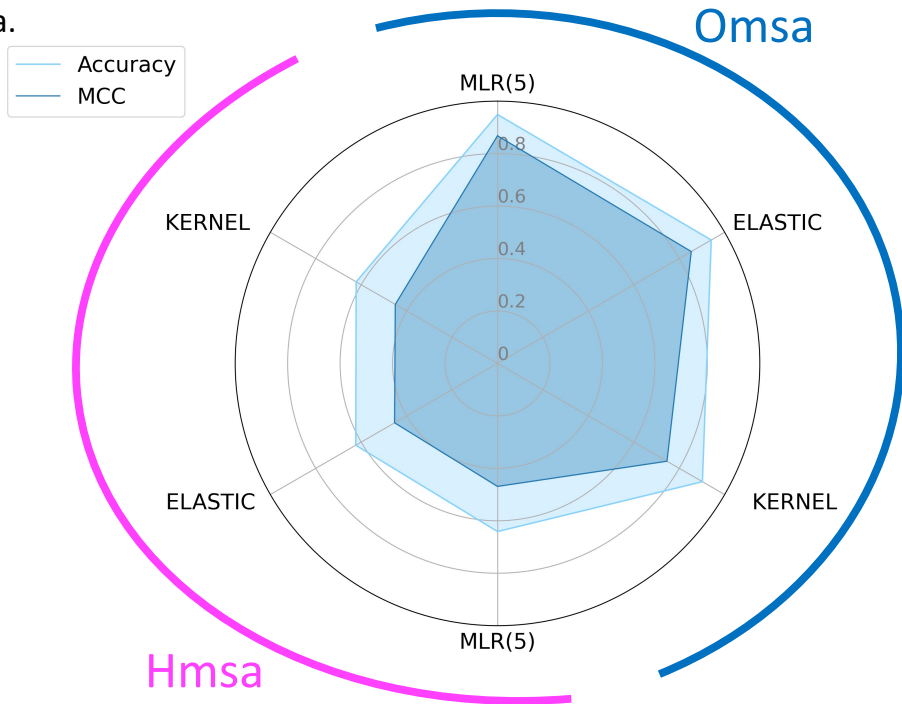

b.

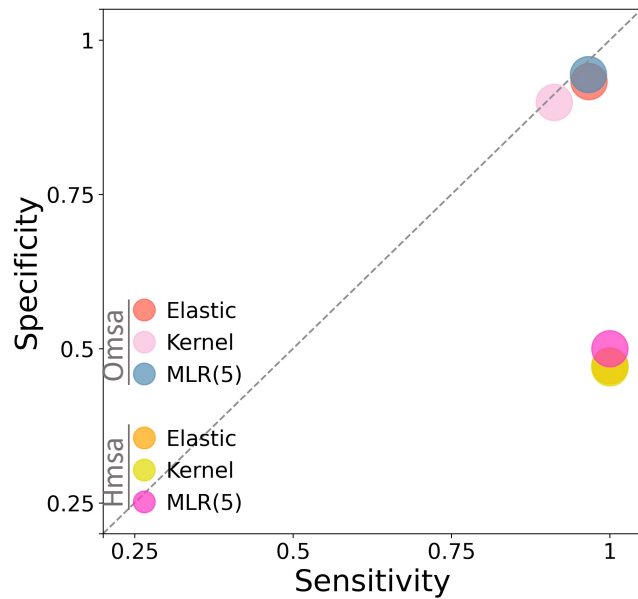

Supplement: Supplementary file 1 [file ijms-22-06226-s001.zip › SUPP_FIGURES_TABLES/SUPPFIGURE8.pdf]

a.

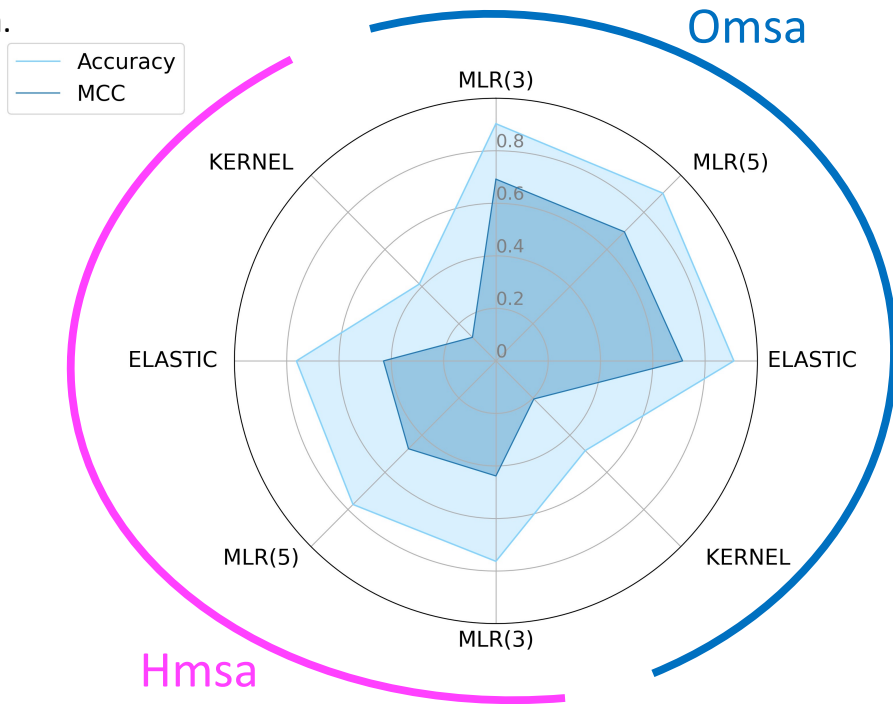

b.

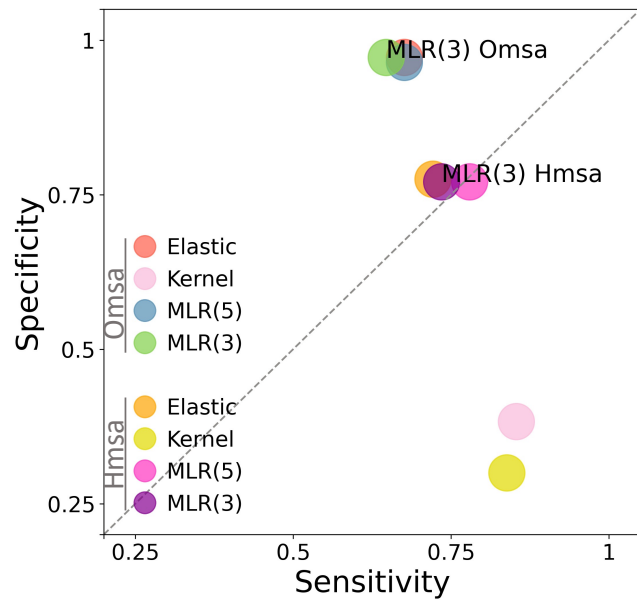

Supplement: Supplementary file 1 [file ijms-22-06226-s001.zip › SUPP_FIGURES_TABLES/SUPPFIGURE9.pdf]
